# The Protective Effects of Human Embryonic Stem Cell-Derived Mesenchymal Stem Cells in Noise-Induced Hearing Loss of Rats

**DOI:** 10.3390/cells11213524

**Published:** 2022-11-07

**Authors:** So Young Kim, Jeoung Eun Lee, Sung Hun Kang, So Min Lee, Jiwon Jeon, Dong Ryul Lee

**Affiliations:** 1 Department of Otorhinolaryngology-Head & Neck Surgery, College of Medicine, CHA University, Seongnam-si 13496, Korea; 2 CHA Advanced Research Institute, Seongnam-si 13488, Korea; 3 School of Medicine, CHA University, Seongnam-si 13488, Korea; 4 Department of Biomedical Science, CHA University, Seongnam-si 13488, Korea

**Keywords:** mesenchymal stem cells, noise, hearing loss, stem cells, cochlea

## Abstract

A few prior animal studies have suggested the transplantation or protective effects of mesenchymal stem cells (MSCs) in noise-induced hearing loss. This study intended to evaluate the fates of administered MSCs in the inner ears and the otoprotective effects of MSCs in the noise-induced hearing loss of rats. Human embryonic stem cell-derived MSCs (ES-MSCs) were systematically administered via the tail vein in adult rats. Eight-week-old Sprague-Dawley rats were randomly allocated to the control (*n* = 8), ES-MSC (*n* = 4), noise (*n* = 8), and ES-MSC+noise (*n* = 10) groups. In ES-MSC and ES-MSC+noise rats, 5 × 10^5^ ES-MSCs were injected via the tail vein. In noise and ES-MSC+noise rats, broadband noise with 115 dB SPL was exposed for 3 h daily for 5 days. The hearing levels were measured using auditory brainstem response (ABR) at 4, 8, 16, and 32 kHz. Cochlear histology was examined using H&E staining and cochlear whole mount immunofluorescence. The presence of human DNA was examined using *Sry* PCR, and the presence of human cytoplasmic protein was examined using STEM121 immunofluorescence staining. The protein expression levels of heat shock protein 70 (HSP70), apoptosis-inducing factor (AIF), poly (ADP-ribose) (PAR), PAR polymerase (PARP), caspase 3, and cleaved caspase 3 were estimated. The ES-MSC rats did not show changes in ABR thresholds following the administration of ES-MSCs. The ES-MSC+ noise rats demonstrated lower ABR thresholds at 4, 8, and 16 kHz than the noise rats. Cochlear spiral ganglial cells and outer hair cells were more preserved in the ES-MSC+ noise rats than in the noise rats. The *Sry* PCR bands were highly detected in lung tissue and less in cochlear tissue of ES-MSC+noise rats. Only a few STEM121-positivities were observed in the spiral ganglial cell area of ES-MSC and ES-MSC+noise rats. The protein levels of AIF, PAR, PARP, caspase 3, and cleaved caspase 3 were lower in the ES-MSC+noise rats than in the noise rats. The systemic injection of ES-MSCs preserved hearing levels and attenuated parthanatos and apoptosis in rats with noise-induced hearing loss. In addition, a tiny number of transplanted ES-MSCs were observed in the spiral ganglial areas.

## 1. Introduction

Noise-induced hearing loss is a type of sensorineural hearing loss that is regularly encountered in clinics and can impose considerable socioeconomic burdens [1]. The primary pathological sites of noise-induced hearing loss are cochlear outer hair cells, followed by spiral ganglial cells and the spiral ligament [2,3]. The oxidative stress and inflammation of cochleae following noise exposure are the main pathophysiological mechanisms of noise-induced hearing loss [4,5]. Myriad signaling cascades have been documented to be evoked after noise exposure and ultimately injure various types of cochlear cells, including outer hair cells, spiral ganglial cells, spiral ligament, stria vascularis and supporting cells [6,7]. Thus, the treatment of noise-induced hearing loss cannot be achieved by targeting a unique molecule or cells. Indeed, many previous preclinical studies have attempted to reverse cochlear injury following noise exposure by the application of antioxidative or anti-inflammatory drugs [8,9], which demonstrated only marginal preservative effects or significant therapeutic effects when administered at mega-doses that cannot be applied in clinics. Regenerative medicine can be an alternative option for overcoming multifocal pathological insults from noise exposure.

A number of prior researchers aimed to restore cochlear function using stem cells [10,11,12]. Because cochlear hair cells and spiral ganglial cells are sensory epithelial cells and peripheral neuronal cells, differentiated stem cells to neural precursor cells were used to regenerate spiral ganglial cells. A few studies reported encouraging results with the differentiation of neural precursor cells to spiral ganglial cells in vitro and in vivo [13]. However, the transplantation efficacy was not sufficient to restore cochlear function and was applied to clinical trials. To enhance the transplantation efficacy of injected stem cells, many studies physically localized the injection sites of stem cells via the round window, posterior semicircular canal and cochlear lateral wall [14,15]. Although local administration of stem cells to the perilymphatic–endolymphatic space of cochleae can reduce the physical distance from the target cell area, the cochlear perilymph or endolymph is a harsh environment for transplanting injected stem cells. In addition, cochlear injection can result in additional injury to residual cochlear function. On the other hand, the homing effects of stem cells have been suggested for systematically administered stem cells following acoustic trauma [16,17]. In addition to transplanted effects, stem cells can act via secretion of trophic factors, including exosomes [18]. Mesenchymal stem cells (MSCs) are known to provide neurotrophins [18]. Thus, a few studies have intended to use exosomes derived from MSCs to restore sensorineural hearing loss. In addition, MSCs can be supposed to be differentiated into neurons [19]. In summary, the systemic application of MSCs can be anticipated for both transplantation and secretion of trophic factors, which can suppress multiple pathologic target pathways of noise-induced hearing loss.

We supposed that the systemic administration of MSCs can be targeted to cochleae with noise insults. In addition, trophic factors from MSCs were supposed to alleviate cochlear injury following noise exposure. To test these roles of MSCs, human embryonic stem cell-derived MSCs (ES-MSCs) were administered to rats via tail vein injection just before noise exposure. We administered ES-MSCs before noise exposure because the deafened cochlea after noise exposure, which induced a permanent threshold shift in this study, is irreversible—hearing function cannot be restored unless the outer hair cells are regenerated. Because previous studies indicated the presence of administered MSCs in spiral ganglial cells but not in outer or inner hair cells, the otoprotective effects of ES-MSCs cannot be anticipated when ES-MSCs are administered following noise exposure. The presence of injected ES-MSCs was evaluated early (a day after ES-MSC injection) and with delay (15 days after ES-MSC injection) to estimate the transplantation of ES-MSCs.

## 2. Results

**Figure 1:**
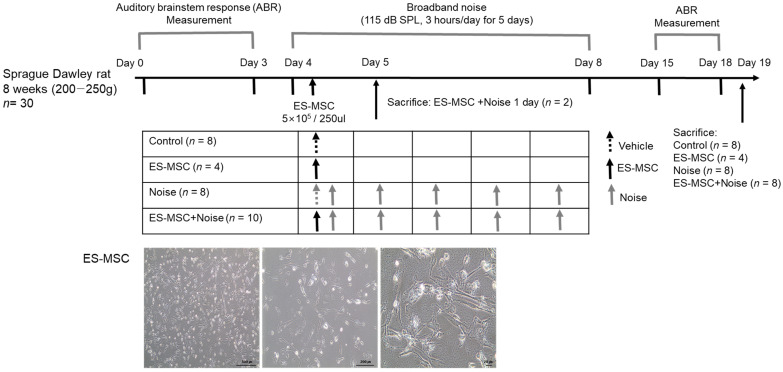
The experimental schedule of the present study. Adult rats were randomly divided into four groups: control, embryonic stem cell-derived mesenchymal stem cells (ES-MSCs), noise, and ES-MSC+noise rats. ES-MSCs were injected at day 4 via the tail vein. Then, broadband noise was exposed for 5 days. The auditory brainstem response (ABR) thresholds were measured before and after treatments. The lower panel showed representative morphologies of the ES-MSCs at different magnifications. The cultured ES-MSCs were injected into rats via the tail vein.

**Figure 2:**
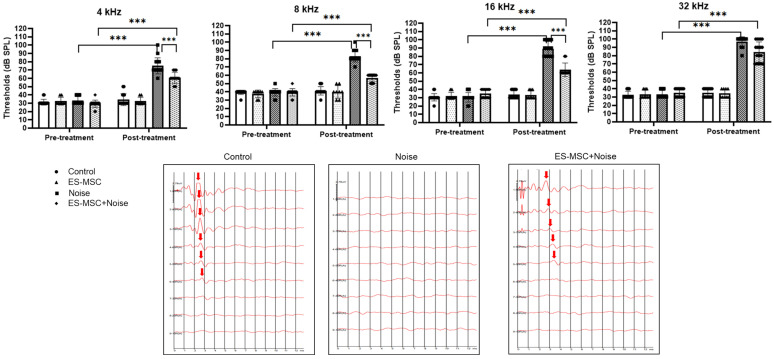
The ABR thresholds before (pretreatment) and after (posttreatment) ES-MSC and noise exposure (*n* = 8 and 16 cochleae for control, noise, and ES-MSC+noise rats and *n* = 4 and 8 cochleae for ES-MSC rats). The ES-MSC + noise rats demonstrated lower ABR thresholds at 4, 8, 16 and 32 kHz. The ABR thresholds were increased after noise exposure. The ES-MSC pretreatment attenuated the ABR threshold shifts at 4, 8 and 16 kHz in the ES-MSC+noise rats. The representative images of ABR in the control, noise, and ES-MSC+noise groups. The red arrow indicated wave II of the evoked potential. (*** *p* < 0.001).

The auditory thresholds were similar among the rats before treatment (pretreatment, Figure 1 and Figure 2). In noise rats, the auditory thresholds were increased at 4, 8, 16 and 32 kHz after noise exposure compared to pre-exposure (post- vs. pre-noise exposure [mean ± SD] = 75.00 ± 9.66 vs. 33.13 ± 4.79 at 4 kHz, 83.13 ± 7.04 vs. 39.38 ± 4.43 at 8 kHz, 89.38 ± 8.54 vs. 30.63 ± 5.74 at 16 kHz and 96.88 ± 6.02 vs. 33.13 ± 4.79 at 32 kHz, all *p* < 0.001). The administration of ES-MSCs did not change the auditory thresholds at 4, 8, 16 and 32 kHz. The ES-MSC + noise rats demonstrated an increased hearing threshold after noise exposure at 4, 8, 16 and 32 kHz (post- vs. pre-noise exposure [mean ± SD] = 60.63 ± 6.80 vs. 29.38 ± 4.43 at 4 kHz, 56.88 ± 4.79 vs. 39.38 ± 4.43 at 8 kHz, 63.75 ± 8.06 vs. 30.63 ± 5.74 at 16 kHz and 84.38 ± 12.09 vs. 35 ± 5.16 at 32 kHz, all *p* < 0.001). However, the hearing thresholds after noise exposure were lower in the ES-MSC + noise rats than in the noise rats at 4, 8 and 16 kHz (ES-MSC+noise rats vs. noise rats [mean ± SD] = 60.63 ± 6.80 vs. 75.00 ± 9.66 at 4 kHz, 56.88 ± 4.79 vs. 83.13 ± 7.04 at 8 kHz, and 63.75 ± 8.06 vs. 89.38 ± 30.63 at 16 kHz, all *p* < 0.001).

**Figure 3:**
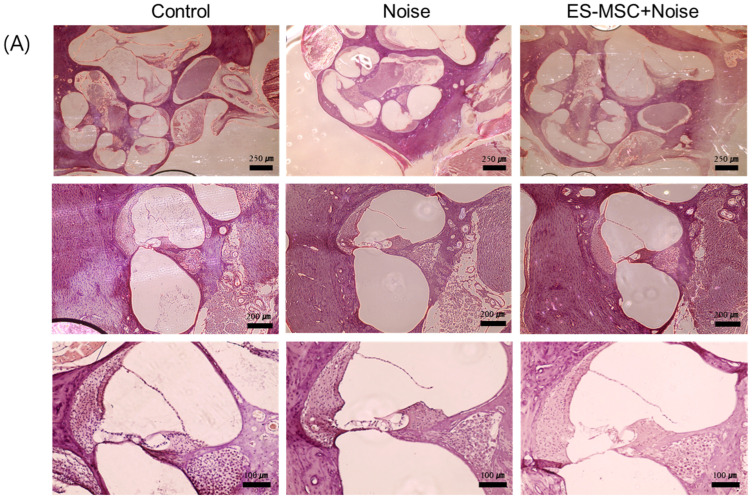

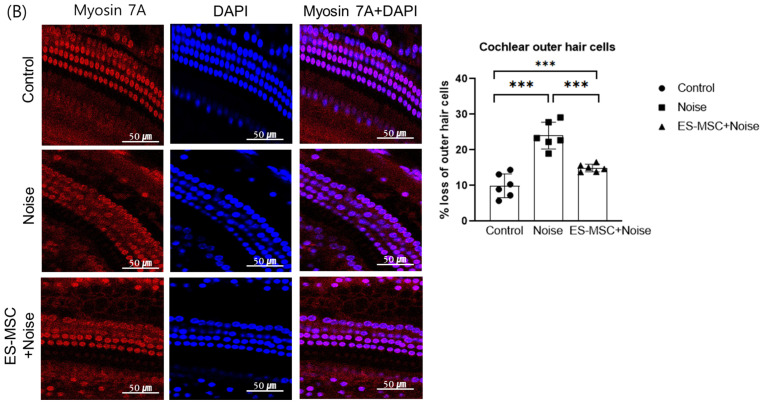
Cochlear histologic findings of the cochleae of control, noise, and ES-MSC+noise rats. (**A**) H&E staining (3 cochleae for each group) demonstrated a greater number of spiral ganglial cells in the ES-MSC+noise rats than in the noise rats which demonstrated gross loss of spiral ganglial cells (**B**) Cochlear whole-mount examinations (6 cochleae for each group) demonstrated a lower proportion of loss of outer hair cells in the ES-MSC+noise rats than in the noise rats. The outer hair cell counts in the middle turn of the cochleae demonstrated lower loss of outer hair cells in the ES-MSC+noise rats than in the noise rats (*** *p* < 0.01, scale bar: (A) 250 µm, 200 µm, 100 µm, (B) 50 µm).

Then, the morphology of the cochleae, including outer hair cells and spiral ganglial cells, was examined in control, noise, and ES+MSC rats (Figure 3). The noise rats demonstrated a sparse number of spiral ganglial cells compared to those of control rats. The ES-MSC+noise rats also showed loss of spiral ganglial cells, while they described greater densities of spiral ganglial cells compared to those of the noise rats. Both the noise rats and the ES-MSC+noise rats demonstrated loss of outer hair cells. To evaluate the degree of loss of outer hair cells, cochlear whole mount examinations were conducted. When outer hair cells were counted in the middle turn of the cochleae, the ES-MSC+noise rats showed less loss of outer hair cells than the noise rats (% loss of outer hair cells in ES-MSC+noise rats vs. noise rats [mean ± SD] = 14.92 ± 1.07 vs. 24.03 ± 3.76, *p* < 0.001).

**Figure 4:**
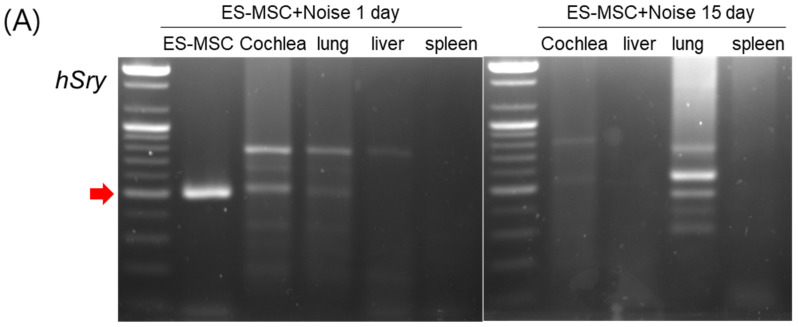

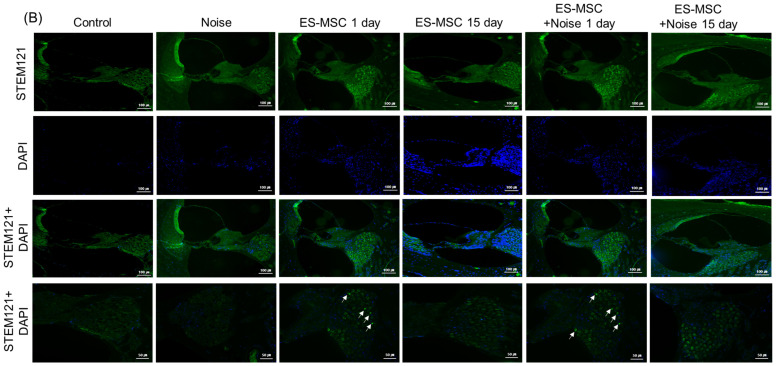
Human DNA detection in ES-MSC+noise rats. (**A**) Human *Sry* gene PCR (2 cochleae or 2 rats for each group) demonstrated *Sry* bands in cochlear and lung tissues 1 day and 15 days after ES-MSC administration. Red arrow indicates the *Sry* band. (**B**) Human cytoplasmic protein examination using STEM121 immunofluorescence staining (2 cochleae for each group) demonstrated a scant number of STEM121-positive cells in the spiral ganglial area in ES-MSC 1-day and ES-MSC+noise 1-day rats. White arrows indicated the STEM121-positivity in the spiral ganglial area. (scale bar (B) 100 µm, 50 µm).

To test the possible transplantation and regeneration of injured cochlea by the injected ES-MSCs, the distributions of injected ES-MSCs were explored using human gene (*sry*) PCR in the cochlea, lung, liver, and spleen (Figure 4). *Sry* bands were detected in the cochlear, lung, and liver tissues of ES-MSC + noise rats one day after ES-MSC administration. At 15 days after ES-MSC injection, the *sry* band was detected in lung tissue, while it was faintly detected in cochlear tissue. The localization of STEM121 (human cytoplasmic protein) was examined in the cochlea to estimate the presence of the injected ES-MSCs in the cochleae. There were no cells reactive to the STEM121 antibody in control and noise rats. In the ES-MSC 1-day and the ES-MSC+noise 1-day rats, there were only a few STEM121-positivities in the spiral ganglial cell area. There were no outer or inner hair cells that were positive for the STEM121 antibody in the ES-MSC or ES-MSC+noise rats.

**Figure 5:**
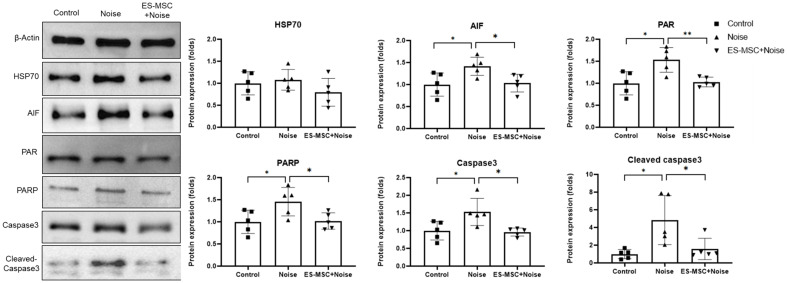
Protein expression levels of heat shock protein 70 (HSP70), apoptosis-inducing factor (AIF), poly (ADP-ribose) (PAR), PAR polymerase (PARP), caspase 3 and cleaved caspase 3 (5 cochleae per group). The protein levels of AIF, PAR, PARP, caspase 3 and cleaved caspase 3 were higher in the noise rats than control rats. These protein levels were lower in the ES-MSC+noise rats than in the noise rats. (* *p* < 0.05, ** *p* < 0.01).

Because there were no implanted ES-MSCs in the outer or inner hair cells, the otoprotective role of secreted molecules, such as HSP70 from exosomes, was expected following noise exposure. Thus, the protein expression levels of HSP70 were estimated in cochlear tissue (Figure 5). However, the protein expression levels of HSP70 were not different among the rats. Because previous studies indicated the activation of AIF/PARP/PAR cascades and apoptosis in noise-exposed models, these protein levels were compared among rats. The cochlear protein expression levels of AIF, PARP, PAR, caspase 3 and cleaved caspase 3 were higher in the noise rats than in the control rats (1.41 ± 0.21-fold, *p* = 0.025 for AIF, 1.45 ± 0.32-fold, *p* = 0.040 for PARP, 1.53 ± 0.28-fold, *p* = 0.015 for PAR, 1.53 ± 0.38, *p* = 0.035 for caspase 3, and 4.84 ± 2.78-fold, *p* = 0.016 for cleaved caspase 3). The ES-MSC+noise rats showed lower protein expression levels of AIF, PARP, PAR and caspase 3 than the noise rats (1.03 ± 0.20-fold, *p* = 0.018 for AIF, 1.02 ± 0.18-fold, *p* = 0.030 for PARP, 1.03 ± 0.11-fold, *p* = 0.006 for PAR, 0.96 ± 0.11, *p* = 0.013 for caspase 3 and 1.58 ± 1.20-fold, *p* = 0.043 for cleaved caspase 3). 

## 3. Discussion

The systemic administration of ES-MSCs attenuated noise-induced hearing loss and cochlear injuries in the rat model in the present study. In addition, the administered ES-MSCs were targeted to the spiral ganglial cell area, although most of the administered cells were located in lung tissue. Thus, the cochlear preservative roles of systematically administered ES-MSCs were not dependent on the transplantation of these cells to injured cochleae. The liberation from parthanatos-mediated and apoptotic cell death may be involved in the otoprotective effects of ES-MSCs. Exosomes secreted from ES-MSCs can mediate the inhibition of cochlear cell death following noise exposure, although the reported secreted protein HSP70 was not highly expressed in ES-MSC-injected rats in this study. The present results enlarged previous findings on the application of MSCs to noise-induced hearing loss by delineating the fates of injected MSCs in cochleae as well as systemic organs. Furthermore, the present findings pave the way to explore the regenerative potential of spiral ganglial cells and spiral ligamental cells by MSCs by documenting the presence of administered cells in these areas among cochlear architecture.

A few previous studies have explored the regeneration or restoration of the cochlea in animals with noise-induced hearing loss using MSCs (Table 1) [16,17,20,21,22]. Three in vivo studies that injected MSCs systemically or locally through round window niche reported the homing of administered MSCs to the injured cochleae following noise exposure [16,17,20]. However, they did not observe hearing improvement associated with MSC treatments [16,17,20]. Previous research described the presence of applied MSCs applied in spiral ganglial cells [20]. However, the number of transplanted MSCs was scant. The current study intended to delineate the acute distribution of applied MSCs and demonstrated protective effects of MSCs in noise-induced hearing loss. Another in vivo study administered MSCs through posterior semicircular canals and documented the rescue of hearing levels following local injection of MSCs [21]. However, they used narrow band noise (16 kHz) with a limited duration of noise exposure (4 h) [21]. Thus, residual cochlear function can be expected in their animal model [21]. In addition, the administered MSCs did not rescue cochlear hair cell injuries [21]. In their RNA-seq analysis, a variety of signaling pathways showed differential expression following MSC administration. Therefore, it can be presumed that the pleiotropic effects of MSCs may exert preservative and recovery effects in noise-induced cochlear injury.

Transplanted ES-MSCs were observed in spiral ganglial cells and spiral ligament cells in this study. The presence of transplanted human bone marrow-derived MSCs has been described in noise-induced hearing loss rats [20]. They presumed that the blood supply of cochlea with fenestrated capillaries in the modiolus portion and non-fenestrated strial capillary endothelial cells may prefer the migration of MSCs from blood to spiral ganglial cells rather than outer hair cells or stria vascularis [20]. In addition to the spiral ganglial cell area, the spiral ligament area also showed the presence of cells positive for human cytoplasmic protein in the present study. In line with the present study, previous studies reported the transdifferentiation of MSCs into spiral ligament fibrocytes [23].

Parthanatos and apoptotic cell death were lower in ES-MSC-injected rats with noise exposure in the present study. Noise exposure induced apoptotic cell death and parthanatos in the cochlea [24,25]. Depending on oxidative stress, which can be induced by noise exposure, AIF can be released and activate PARP and accumulate PAR, resulting in cell death referred to as parthanatos [26]. A previous study reported the protective effects of HSPs secreted from MSCs in cochlear injuries [27]. However, the expression level of HSP70 was not higher in the ES-MSC treated rats in the present study. Thus, other mechanisms may act on hearing preservation from acoustic trauma. The otoprotective effects of ES-MSCs may not be dependent on a single or a few mediators. Instead, a large number of mediators and signaling cascades can be involved in the otoprotective effects of ES-MSCs. A prior study reported that genes involved in immune modulation, hypoxia response, mitochondrial function, and regulation of apoptosis were upregulated in MSC-injected mice with noise-induced hearing loss [21]. Thus, MSCs may mediate otoprotective effects by regulating a variety of signaling pathways, including parthanatos and apoptosis. In addition, neurotrophins, such as brain-derived nerve growth factor (BDNF), secreted from MSCs can exert protective roles in spiral ganglial cells. MSCs have been reported to secret neurotrophins including BDNF, to attenuate neural injuries [28,29]. Because the administration of neurotrophin recovered cochlear injuries in prior research [30,31], MSC-derived neurotrophin can mediate the otoprotective effects in the current research. Further study may be warranted to identify a number of key mediators of hearing preservation.

Although significant hearing preservation was noted in the ES-MSC-injected rats, hearing loss from noise was not totally prevented in the ES-MSC-injected rats in this study. In particular, the high frequency hearing level was not preserved in the ES-MSC-injected rats. The otoacoustic emission test and ABR measure for other frequency, such as 24 kHz can elaborate the detailed hearing preservation effects of E-MSCs, which were not conducted in the present study. Outer hair cell counting was conducted in the middle turn of cochleae. In addition, the human genomic DNA and human cytoplasmic proteins were faded out from the cochleae over time. The presence or transplantation of administered ES-MSCs cannot be surely determined using human DNA detection or observation of human cytoplasmic protein as described in the present study. Moreover, the characteristics of potentially transplanted cells need to be unraveled in further analyses. In the present study, 5 × 10^5^ cells were administered based on previous studies that used cells with a range from 1 × 10^5^ to 4 × 10^6^ in adult rats (Table 1). However, this study did not examine dose-dependent results, and further consideration may be required for the optimal dose of MSCs. To overcome these limitations, the repetitive administration of ES-MSCs may be one solution. If this is not available due to the restriction of ES-MSCs, adjuvant therapy can be tried in clinics. For instance, chemotactic agents, such as CXC chemokine, or neurotrophic factors can be added in combination with the administration of ES-MSCs [32]. In addition, the immune response can attack the applied ES-MSCs, which decreased transplantation in the cochleae in this study. Although the clinical application of ES-MSCs can be limited due to the possible harms from immune reactions, uncontrolled proliferation or oncogenic potential and ethical issues, autologous MSCs can be harvested from various organs, including bone marrow or skin [20,33]. In addition, the active components, such as exosomes, can be extracted from ES-MSCs for clinical use to rescue cochleae from hearing loss. Because there is no therapeutic option for sensorineural hearing loss, including noise-induced hearing loss, the application of MSCs may be a promising choice for restoring sensorineural hearing loss; moreover, its shortcomings can be overcome in the near future.

## 4. Materials and Methods

### 4.1. Ethical Approval

All experimental procedures were conducted following institutional approval from the Institutional Animal Care and Use Committee of CHA University Medical School (IACUC220002).

### 4.2. Preparation of Human Embryonic Stem-Derived Mesenchymal Stem Cells (ES-MSCs)

Human ES-MSCs were obtained from CHA Advanced Research Institute as previously published [34,35,36,37]. ES-MSCs were derived from human embryonic stem cells (CHA-hES 15; Korea Stem Cell Registry code hES12010028) by the 2D direct differentiation technique [37]. The MSC characteristics and genetic stability of ES-MSCs were confirmed in a previous study [37]. ES-MSCs were cultured in DMEM/F12 media (Gibco, Loughborough, Leicestershire, England, 11320-033) with 10% fetal bovine serum (Gibco, Loughborough, Leicestershire, England, 16000-044), 1% nonessential amino acids (Gibco, Loughborough, Leicestershire, England, 11140-050), 1% penicillin/streptomycin (Gibco, Loughborough, Leicestershire, England, 15140-122) and 0.1% β-mercaptoethanol (Gibco, Loughborough, Leicestershire, England, 21985-023). ES-MSCs at passages 9–10 were transplanted into rats. The ES-MSCs were separated from the culture dish using 0.05% trypsin/EDTA and centrifuged three times. The cell pellets were resuspended with DPBS. ES-MSCs (5 × 10^5^/250 µL) were transplanted into rats via tail vein injection.

### 4.3. ES-MSC Injection and Noise Exposure

Eight-week-old Sprague-Dawley rats were randomly classified into four groups (*n* = 30). The control rats were injected with 250 µL of saline via the tail vein on day 4 and raised in background noise (30–50 dB SPL). The ES-MSC rats were injected with 5 × 10^5^/250 µL of ES-MSCs via the tail vein on day 4 and raised in background noise (30–50 dB SPL). The noise rats were injected with 250 µL of saline via the tail vein on day 4 and exposed to broadband noise (2–20 kHz) with 115 dB SPL for 3 h daily for 5 days from days 4–8. The ES-MSC + noise rats were injected with 5 × 10^5^/250 µL of ES-MSCs via the tail vein on day 4 and exposed to broadband noise (2–20 kHz) with 115 dB SPL for 3 h daily for 5 days from days 4–8. Noise exposure was conducted within 1 h following ES-MSC or saline injection. Two rats in the ES-MSC+noise group were sacrificed on day 5 to evaluate the distribution of injected ES-MSCs (ES-MSC+noise 1 day).

### 4.4. Examination of Auditory Evoked Potentials

The auditory thresholds were measured at days 0–3 and days 15–18 using a SmartEP system (SmartEP Intelligent Hearing System, Miami, FL, USA) [38]. The auditory brainstem response (ABR) was examined at 4, 8, 16 and 32 kHz. Rats were anesthetized using 40 mg/kg zoletil and 10 mg/kg xylazine. The electrodes were inserted in the vertex (reference), contralateral thigh (ground), and ipsilateral retro-auricular space (measuring). The auditory stimuli were transmitted through an EC1 electrostatic speaker and a plastic earphone fitted into the external auditory canal. Pure tones with frequencies of 4, 8, 16 and 32 kHz had a duration of 1562 us, a Blackman envelope, a stimulation rate of 21.1/s and 1024 sweeps. The sound amplitude was measured from 90 dB SPL to 20 dB SPL with 10 dB SPL lowering. The hearing threshold was designated as 100 dB SPL, when there is no evoked potential at 90 dB SPL. The lowest sound amplitude with wave II was defined as the auditory threshold [39].

### 4.5. Examination of Cochlear Histology

To evaluate cochlear morphology, hematoxylin and eosin (H&E) staining was conducted using paraffin sections of the cochlea. Three cochleae per group were examined for H&E staining. After dipping in 4% paraformaldehyde solution, the cochleae were decalcified for 7–14 days. The membranous labyrinth was immersed in a paraffin solution. Then, the paraffin block was sliced into 10 µm sections using a microtome. The sections were deparaffinized and washed in ethanol and phosphate-buffered saline. The sections were incubated with hematoxylin for five minutes and eosin for 45 s. The cochleae were examined under the EVOS^TM^ XL Core Imaging System (Invitrogen, Carlsbad, CA, USA, #AMEX1000).

Another six cochleae per group were examined for cochlear whole mounting to evaluate the outer hair cell integrity. Following fixation and decalcification of the bony labyrinth, cochlear outer hair cells were dissected under a microscope. Then, 10% goat serum was used for blocking. Then, anti-myosin 7A (Santa Cruz, Dallas, TX, USA, Sc74516) was added and incubated overnight at 4 °C. Following washing three times, Alexa 594 anti-mouse IgG (Abcam, Cambridge, MA, USA, ab150108) and 4’,6-diamidino-2-phenylindole dihydrochloride (DAPI) were added and incubated for three hours at room temperature. The outer hair cells were inspected under a confocal microscope (Zeiss LSM 880, Zeiss, Oberkochen, Land Baden-Württemberg, Germany). In the middle turn of the cochleae, myosin 7A reactive cells were calculated, and the percentage of loss of outer hair cells was estimated as previously described. Three rows of outer hair cells were observed for the missing of hair cells [40]. The absence of hair cells was considered when there was a gap in the outer hair cell row or there was no apparent nucleus.

### 4.6. Distributions of Injected Human ES-MSCs

To estimate the fates of injected ES-MSCs in rats, the presence of human genomic DNA was examined using human *Sry* PCR, and the presence of human cytoplasmic protein was examined using human STEM121 antibody. Two mice per group were examined for human *Sry* PCR in cochlear, lung, liver and spleen tissues. Genomic DNA was extracted using a Solg™ Genomic DNA Prep kit (SolGent, SGD41-C100). Genomic DNA (3 µL) was mixed with 1 µL of forward primer (human *Sry*-F: GTAAAGGCAACGTCCAGGATAGAG), 1 µL of reverse primer (human *Sry*-R: GCATCTAGGTAGGTCTTTGTAGCC) and AccuPower PCR premix (Bioneer, K-2012). PCR was conducted using a LifeTouch Thermal Cycler (Hangzhou Bioer Technology). The cycling conditions comprised an initial denaturation step at 95 °C for 3 min, 30 cycles of 30 s denaturation at 95 °C, 30 s annealing at 60 °C and 30 s extension at 72 °C, followed by a final extension at 72 °C for 5 min [35]. For the second round of amplification, 1 µL of amplified product was thermally cycled. The initial denaturation was at 95 °C for 2 min, followed by 30 cycles of amplification and a final extension [35]. The amplified products were electrophoresed in a 1.5% agarose gel.

Two cochleae per group were examined for the presence of human cytoplasmic STEM121. The cochleae were sectioned, and slides were used for H&E staining and STEM121 staining. The prepared cochlear paraffin sections were incubated with STEM121 mouse monoclonal antibody IgG1 (TaKaRa, Y40410, Shiga, Japan) overnight at 4 °C. Following washing three times, Alexa 488 anti-mouse Ig (ThermoFisher, Waltham, MA, USA, A21200) and 4’,6-diamidino-2-phenylindole dihydrochloride (DAPI) were added for three hours at room temperature. The presence of STEM121 reactive cells was inspected under a fluorescence microscope (Carl Zeiss, Axiovert 200 M, Oberkochen, Germany).

### 4.7. Expression Levels of Heat Shock Protein 70 (HSP70) and Apoptosis and Parthanatos-Related Markers

Five cochleae per group were examined for the protein expression levels of HSP70, apoptosis-inducing factor (AIF), poly (ADP-ribose) (PAR), PAR polymerase (PARP) and caspase 3. Protein was extracted from the harvested tissues using a PRO-PREP kit (Intron Technology). The quantities of extracted proteins were estimated using BCA Protein Assay Reagents (Thermo Fisher Scientific, Waltham, MA, USA). Sodium dodecyl sulfate–polyacrylamide gel electrophoresis was conducted in an 8% gel at 80 V for 90 min. Then, the gels were transferred to polyvinylidene difluoride membranes (Merck Millipore, Burlington, MA, USA) at 300 mA for 90 min. The membranes were treated with 1:1000 anti-rabbit IgG HSP70 (Invitrogen, PA5-28003), anti-rabbit AIF (Cell Signaling Technology, 4642S), anti-mouse IgG3 PAR (ENZO Life Sciences, ALX-804-220-R100), anti-rabbit PARP (Cell Signaling Technology, 9542S), anti-rabbit caspase 3 (Cell Signaling Technology, 9662S), anti-rabbit cleaved caspase 3 (Cell Signaling Technology, 9662s) and anti-rabbit monoclonal β-actin (Cell Signaling Technology, Danvers, MA, USA, D6A8) overnight at 4 °C. The secondary antibodies (anti-rabbit IgG, HRP-linked; Cell Signaling Technology, #7074S and goat anti-mouse IgG H&L [HRP]; Abcam, #ab97023) were added for two hours at room temperature. The bands were detected using an enhanced chemiluminescence kit (Bio-Rad, Hercules, CA, USA). Quantification was conducted using ImageJ software version. 1.53k (National Institutes of Health, Bethesda, MD, USA). The measured values were normalized based on the values of β-actin and then compared to the values of control rats.

### 4.8. Statistical Analysis

The auditory thresholds before and after treatments were analyzed using a paired *t* test. The auditory thresholds, the percentage of loss of outer hair cells, and protein expression levels were compared between groups using an unpaired *t* test. *p* < 0.05 was regarded as statistically significant. All values were graphed and described as the means with standard deviation (SD, ±). SPSS version 21.0 (IBM Corp., Armonk, NY, USA) was used in the present study.

## 5. Conclusions

The systemic application of ES-MSCs has preservative effects on noise-induced cochlear injuries of the apex-middle region. The functional and histological protection of the cochleae was accompanied by a reduction in cochlear cell death via parthanatos and apoptosis. Both the transplantation and secretary function of ES-MSCs may contribute to otoprotective effects in noise-induced hearing loss.

## Figures and Tables

**Table 1:** In vivo studies of mesenchymal stem cells (MSCs) in noise-induced hearing loss.

Author [Year]	Species	Type of Mesenchymal Stem Cells (MSCs)	Noise Exposure	Route of Delivery	Dosage (Cells)	Time	Outcome
SY Kim et al. [2022]	SD rat (8 weeks)	Human ES-MSCs	115 dB SPL, 3 h/day for 5 days	Tail vein	5 × 10^5^	pretreatment	4, 8, 16 kHz improved
Athanasia Warnecke et al. [2021]	C57BL/6 mice (4 weeks)	Human WJ-MSCs	118 dB SPL, 4 h	PSC	1 × 10^3^	posttreatment (48 h)	4, 8, 32 kHz improved
Liangwei Xu et al. [2020]	Bama minipig (4 weeks)	Human WJ-MSCs	80 times continuous impulse noise	Subarachnoid cavity	1 × 10^8^	posttreatment (1 weeks)	1, 2, 4, 8, 16, 24 kHz improved
Ali Asghar Peyvandi et al. [2017]	Wistar rat (12 weeks)	Rat BM-MSCs	115 dB SPL, 6 h/day for 5 days	RW niche	1 × 10^5^	posttreatment	DFO pre-conditioning increases homing ability of MSCs into injured ear
Ali Asghar Peyvandi et al. [2017]	Wistar rat (8–12 weeks)	Rat BM-MSCs	110 dB SPL, 6 h/day for 5 days	RW niche	1 × 10^5^	posttreatment	CXCR4 antagonist (AMD3100) decreases homing ability of MSCs into injured ear
BY Choi et al. [2012]	SD rat (7–8 weeks)	Human BM-MSCs	120 dB SPL, 9 h	Tail vein	4 × 10^6^	posttreatment (48 h)	No statistically significant difference

ES-MSC, Embryonic stem cell-derived MSC; WJ-MSC, Wharton’s jelly-derived MSC; BM-MSC, bone marrow-derived MSC; PSC, posterior semicircular canal; RW niche, round window niche; DFO, deferoxamine; CXCR4, CXC chemokine receptor-4; SPL, sound pressure level.

## Data Availability

The data presented in this study are available upon request from the corresponding author.
